# Hippocampal CA1 βCaMKII mediates neuroinflammatory responses via COX-2/PGE2 signaling pathways in depression

**DOI:** 10.1186/s12974-018-1377-0

**Published:** 2018-12-08

**Authors:** Qiqi Song, Cuiqin Fan, Peng Wang, Ye Li, Mu Yang, Shu Yan Yu

**Affiliations:** 1 Department of Physiology, School of Basic Medical Sciences, Shandong University, 44 Wenhuaxilu Road, Jinan, 250012 Shandong Province People’s Republic of China; 2 Shandong Provincial Key Laboratory of Mental Disorders, School of Basic Medical Sciences, 44 Wenhuaxilu Road, Jinan, 250012 Shandong Province People’s Republic of China

**Keywords:** βCaMKII, p38 MAPK, PGE2, Depression, CA1 hippocampus

## Abstract

**Background:**

Neuroinflammation has recently emerged as a critical risk factor in the pathophysiology of depression. However, the underlying molecular mechanisms and the development of novel therapeutic strategies as means to target these inflammatory pathways for use in the treatment of depression remain unresolved. In the present study, we aimed to investigate the molecular events of neuroinflammation as related to its induction of depression-like behaviors.

**Methods:**

Chronic unpredictable mild stress (CUMS) or lipopolysaccharide (LPS) was used to induce depression-like behaviors in rats. The inflammatory factors and related proteins were verified by RT-PCR, immunoblotting, and immunofluorescence assay. In vivo intracerebral injection of adenovirus-associated virus (AAV) in rats was used to overexpress or block the function of the β form of the calcium/calmodulin-dependent protein kinase type II (βCaMKII). In vivo intracerebroventricular injection of SB203580 was used to block p38 mitogen-activated protein kinase (MAPK). Finally, the prostaglandin E2 (PGE2) concentration was verified by using enzyme-linked assay kit.

**Results:**

The expression of cyclo-oxygenase (COX)-2, which is responsible for production of the pro-inflammatory factor PGE2 and thus glial activation, was increased in the CA1 hippocampus in a rat model of depression. Further, the βCaMKII in CA1 was significantly upregulated in depressed rats, while antidepressant treatment downregulated this kinase. Overexpression of βCaMKII via infusion of a constructed AAV-βCaMKII into the CA1 region resulted in phosphorylation of the p38 MAPK and the activating transcription factor 2 (ATF2). These effects were accompanied by an enhanced activity of the COX-2/PGE2 pathway and effectively induced core symptoms of depression. Conversely, knockdown of βCaMKII within the CA1 region reversed these inflammation-related biochemical parameters and significantly rescued depression symptoms.

**Conclusion:**

These results demonstrate that βCaMKII can act as a critical regulator in depression via activating neuroinflammatory pathways within the CA1 region. Moreover, this study provides new perspectives on molecular targets and drug therapies for future investigation in the treatment of depression.

**Electronic supplementary material:**

The online version of this article (10.1186/s12974-018-1377-0) contains supplementary material, which is available to authorized users.

## Background

Major depression disorder (MDD) is one of the most prevalent psychiatric disorders. Current hypotheses regarding the basis for MDD have focused on the concept that it is frequently associated with the occurrence of neuroinflammation within specific brain regions in response to external stress stimuli. Despite recent advances in the treatment of depression that have offered improvements in behavioral responses and reductions in the extent of relevant neurobiological deficits in MDD patients, much work remains for the understanding and management of this disorder. For example, current clinical treatments, such as administration of classical monoaminergic antidepressants and some anti-inflammatory drugs, are often only marginally effective and possess many side effects [1]. Therefore, the identification of novel inflammatory system targets as therapeutic strategies for the treatment of psychiatric disorders, like MDD, represents an essential area of current investigation.

Accumulating evidence has revealed that critical interactions exist between immune activation and changes in brain circuits as related to mood and behavioral disorders. Within the central nervous system (CNS), immune responses to stressful stimuli are characterized by a rapid activation of microglial cells and astrocytes and release of pro-inflammatory cytokines, including interleukin-1β (IL-1β), tumor necrosis factor (TNF)-α, and prostaglandin E2 (PGE2). Results from clinical studies have indicated that these inflammatory markers are frequently maintained at persistently elevated levels in patients with MDD and are often associated with the duration or severity of the mood disorder [2]. Such observations support the significance of preventing the release of pro-inflammatory cytokines as a potential therapeutic approach to improve depressive symptoms and stress resilience.

Results from previous studies have shown that the Ca^2+^-triggered activation of calcium/calmodulin-dependent protein kinases type II (CaMKIIs) are related to stress-induced depressive symptoms [3–6]. Moreover, inhibition of CaMKII attenuates inflammatory-associated protein kinases and mediators in rat cerebrovascular inflammation [7]. Within spinal neurons, CaMKII acts as an upstream cascade to facilitate cyclo-oxygenase (COX)-2 transcription and expression, which is involved in the development and/or maintenance of inflammatory pain [8]. COX-2 enzymatic hyperactivity can prompt production of the pro-inflammatory factor, PGE2. However, with regard to depression, the specific isoforms of CaMKIIs, as well as specific brain regions where this may function, have yet to be identified. Recent findings have revealed that βCaMKII is present in the lateral habenula, an area which serves as a key determinant for neuronal activity and behavioral phenotypes of depression [9]. Therefore, these data suggest that βCaMKII activation appears to afford a critical link between stress exposure and immune activation in patients with MDD, both of which are key factors in the pathogenesis of this psychiatric disorder. What is not known of this link are the molecular mechanisms, in particular whether changes in CA1 hippocampal CaMKIIs are involved in the pathophysiological mechanisms of depression. Therefore, a major goal of this study was to focus on the βCaMΚΙΙ-activated signaling pathway as a basis for improving neuroinflammation resilience and depressive symptoms.

An additional factor that has been associated with a number of developmental and pathological conditions including multiple neurodegenerative pathologies is activating transcription factor 2 (ATF2) [10]. ATF2 can act as a critical factor in inflammation-related signaling pathways, as indicated by the findings that alterations in expression and activity of ATF2 are linked to inflammation-related pathologies [11]. However, whether ATF2 functions as a downstream molecule of βCaMΚΙΙ to upregulate COX-2 transcription and hence facilitate inflammatory responses related to depression remains unknown.

Based on this background information, in the present study, we investigated whether dysregulation of βCaMKII, a process that is linked to activation of COX2/PGE2-driven inflammatory pathways is involved in the genesis and progression of depression. Such findings would significantly help to clarify the relevant neurobiological alterations that drive the behavioral symptoms displayed in depression. Moreover, they would identify the potential roles of βCaMKII which could be targeted for the development of more effective and novel therapeutic strategies in the treatment of depression.

## Methods

### Animals

Male Wistar rats (160–180 g) were obtained from the Shandong University Experimental Animal Centre. All animal procedures were performed in accordance with the International Guiding Principles for Animal Research provided by the International Organizations of Medical Sciences (CIOMS) Council and were approved by the Animal Care and Use Committee of Shandong University. Rats were housed in groups of four per cage under controlled temperature (22–24 °C) and light (12-h light/dark cycle) conditions and were acclimatized to this laboratory environment for at least 1 week prior to experimental procedures and then housed individually in a separate room to subject chronic stress regimes for 5 weeks. All efforts were made to minimize pain and numbers of the animals used in these experiments.

### Reagents

Fluoxetine, celecoxib, SB203580, lipopolysaccharide (LPS), and dimethyl sulfoxide (DMSO) were purchased from Sigma-Aldrich (St. Louis, MO, USA). Polyclonal rabbit anti-ionized calcium-binding adaptor molecule-1 (Iba-1) (#019-19741) was purchased from Wako Pure Chemical Inc. (Japan). Polyclonal rabbit anti-glial fibrillary acidic protein (GFAP) (60190-1-Ig) was purchased from the Proteintech Group (Rosemont, IL, USA). Polyclonal rabbit anti-βCaMKII (ab34703) and polyclonal goat anti-4-hidroxynonenal (4-HNE, ab46545) were purchased from Abcam (Cambridge, UK). Polyclonal rabbit anti-p38 MAPK (CST-9212), anti-phospho-p38 MAPK (CST-9211), anti-phospho-CREB (CST-9198), anti-phospho-ATF-2 (CST-9221), rabbit anti-NeuN (CST-24307), anti-β-actin (CST-4970), and monoclonal rabbit anti-COX2 (CST-12282) were purchased from Cell Signaling (Beverly, MA, USA). Rhodamine (TRITC)-conjugated goat anti-rabbit IgG and fluorescein (FITC)-conjugated Affinipure goat anti-mouse IgG were purchased from the Proteintech Group. Peroxidase-conjugated goat anti-rabbit/mouse IgG was purchased from Zhongshan Golden Bridge Biotechnology. The rat PGE2 ELISA kit was purchased from the Enzyme-linked Biotechnology Co (Shanghai, China). Constructed adeno-associated virus pHBAAV-r-βCaMKII-GFP (AAV-βCaMKII) (#HY20170713GX-AAV03) and pHBAAV-r-βCaMKII shRNAi-GFP (AAV-βRNAi) (#HY20170713GX-AAV04) were purchased from Hanbio Biotechnology Co. (Shanghai, China).

### Depression animal model

#### CUMS model

**Fig. 1:**
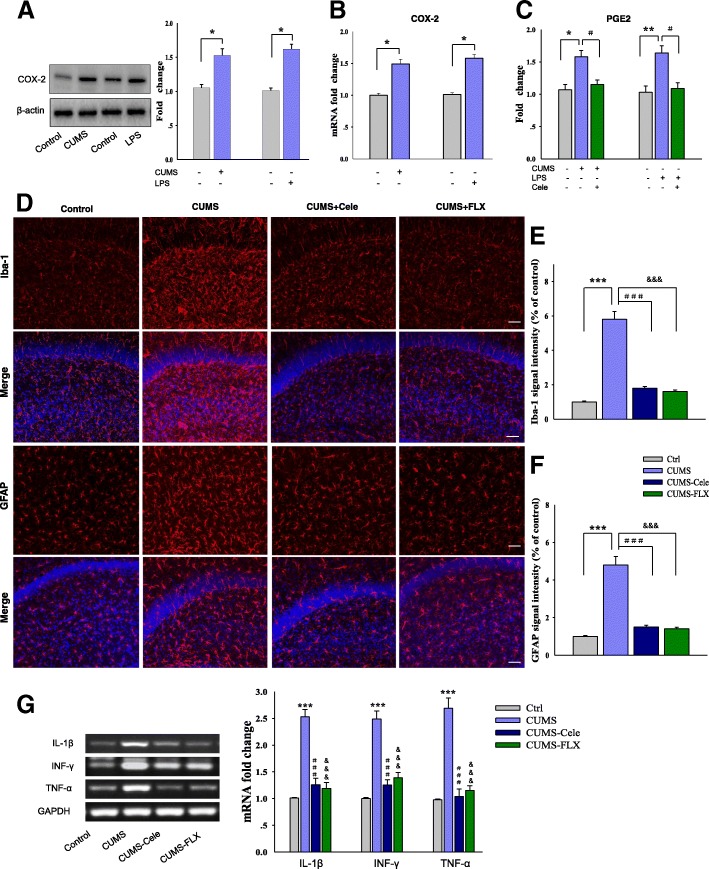
COX-2 mediated inflammatory responses in the CA1 hippocampus in a rat model of depression. **a** Western blot analysis showing upregulation of COX-2 protein in CA1 of depressed rats. **b** QPCR analysis of COX-2 mRNA in CA1 of depressed rats. **c** Content assays showing expressions of PGE2 in CA1 of depressed rats. **d** Immunofluorescence signals of Iba1positive microglial cells and GFAP-positive astrocytes within the CA1 region. Nuclei (blue) are stained with DAPI. Scale bar is 50 μm. **e** Quantification of Iba1-positive cell numbers in CA1 regions. **f** Quantification of GFAP-positive cell numbers in CA1 regions. **g** RT-PCR assays of mRNA expression levels of IL1β, TNF-α, and IFN-γ within the CA1 regions of rats. *N* = 6 per group. Data are presented as the means ± SEM. *P < 0.05, **P < 0.01, ***P < 0.001 CUMS (or LPS) vs control group; ^#^*P* < 0.05, ^##^*P* < 0.01, ^###^*P* < 0.001 CUMS (or LPS)+Cele vs depression group; ^&^*P* < 0.05, ^&&^*P* < 0.01, ^&&&^*P* < 0.001 CUMS (or LPS)+FLX vs depression group (Cele, celecoxib; FLX, fluoxetine)

The chronic unpredicted mild stress (CUMS) procedure was used for induction of depression in a rat model with minor modifications as described previously [12]. Briefly, rats were housed individually in a separate room and subjected to a variety of daily stress regimes for 5 weeks. Stressors included 24-h food deprivation followed by 24-h water deprivation, 45° cage tilt (24 h), 5-min cold swimming (at 4 °C), wet bedding (24 h), foot shock (0.5 mA, 0.5 s), physical restraint (2 h), cage shaking (2 h), and overnight illumination. Stressors were applied daily to each rat in a random and unpredictable order (Fig. 1).

#### LPS-induced model

The LPS-induced depression animal model was constructed with minor modifications as described previously [13, 14]. LPS (0.5 mg/kg) was intraperitoneally (i.p.) injected each day for 1 week to induce depression-like behaviors in rat (Additional file 1: Figure S1A).

### Drug treatment

Fluoxetine was dissolved in sterile endotoxin-free saline (NaCl, 0.9%) at a concentration of 10 mg/ml. Celecoxib was dissolved in DMSO (0.1%) at a concentration of 10 mg/ml. The p38-MAPK antagonist SB203580 was dissolved in DMSO (0.1%) at a concentration of 0.25 μg/μl. LPS was dissolved in 0.9% saline at a concentration of 10 mg/ml. Rats were randomly allocated to one of the following groups (*N* = 18/group): (a) control (non-stressed group), (b) CUMS, (c) LPS (0.5 mg/kg), (d) CUMS treated with celecoxib (20 mg/kg) (CUMS+celecoxib), (e) CUMS treated with fluoxetine (40 mg/kg) (CUMS+FLX), (f) LPS treated with celecoxib (LPS+celecoxib), (g) LPS treated with fluoxetine (LPS+FLX), (h) SB203580 (10 μg/kg) treatment followed by CUMS (SB203580+CUMS), and (i) DMSO (0.1%) treatment followed by CUMS (DMSO+CUMS). In all experiments, celecoxib, fluoxetine, LPS, and DMSO were administered via an intraperitoneal (i.p.) injection while SB203580 was administered intracerebroventricularly (i.c.v.) at 60 min prior to CUMS procedure daily for 5 weeks (Additional file 1: Figure S1B).

### Behavioral tests

Behavioral tests were conducted after the 5 weeks of CUMS exposure. Scoring of these tests was performed by an observer blind to the experimental treatment.

#### Sucrose preference test (SPT)

The sucrose preference test was conducted as described previously with minor modifications [12]. In the adaption phase, rats were placed individually in cages with two bottles of sucrose solution (1%, *w*/*v*) for the first 24-h period. In the second 24-h period, one bottle of sucrose solution was then replaced with a bottle containing tap water. In the test phase, rats were deprived of water and food for 24 h and then permitted access to the two bottles for 3 h, one containing 100 ml of 1% sucrose solution and the other containing 100 ml of tap water. The sucrose preference was defined as sucrose consumption/[water consumption + sucrose consumption] × 100% during the 3-h test.

#### Forced swim test (FST)

One day after the sucrose preference test, the forced swim test was conducted as described previously [15]. Briefly, in the training session, rats were placed individually in a cylinder of water (height 80 cm, diameter 30 cm, temperature 25 °C) for 15 min of forced swimming. In the test session that was performed 24 h later, rats were individually placed in a cylinder for a 5-min period. The immobility time and swimming time of each rat were recorded during this test. The immobility time was defined as floating with only limited movements to maintain their head above water.

### Stereotaxic injection of the AAV virus

After behavioral test, part of the rats from the non-stressed group and depressed group were randomly allocated to one of the following six groups (*N* = 18/group): (a) wild type (non-stressed and non-injected group), (b) wild type + AAV-control (GFP-Cre construct), (c) wild type + AAV-βCaMKII, (d) stressed, (e) stressed + AAV-control (GFP-Cre construct), and (f) stressed + AAV-βRNAi. For viral injections, rats were anesthetized with sodium pentobarbital (150 mg/kg, i.p.) and placed in a stereotaxic frame. Rats were infused bilaterally with 1–1.5 μl of purified and concentrated AAV virus (~ 10^12^ infection units per ml) into the hippocampus CA1 region (coordinates from the bregma, − 3.48 mm; medial/lateral, ± 1.8 mm; dorsal/ventral, − 2.55 mm, as established for Wistar rats at 8 weeks of age) using an electric microinjection pump (Stoelting, USA) at a rate of 150 nl/min. Behavioral experiments or biochemical assays were performed at > 10 days after injection. Injection sites were verified after behavioral tests, and only the rats with correct injection sites were used for the following assays. Each CA1 region was sectioned into six serial slices (30 μm) and examined under fluorescent microscopy for counting of infected cells. The total number of neurons was averaged from counting NeuN signals in serial sections of three control rats. Infection rates were presented as number of infected cells/number of total neurons.

### Intracerebroventricular injection

For intracerebroventricular injection of SB203580, part of rats from the depressed group were anesthetized with 2.5% isoflurane and placed in the stereotaxic apparatus. A portion of the parietal skull was carefully removed, and a guide cannula was then inserted into the right lateral ventricle (coordinates from the bregma, − 1.5 mm; medial/lateral, ± 1.0 mm; dorsal/ventral, − 3.2 mm) and fixed with dental cement. To verify the injected location, the guide cannula was connected to a polyethylene (PE) pipe that was filled with artificial cerebrospinal fluid. Then the PE pipe was vertically inserted into the right lateral ventricle through the guide cannula slowly. While the liquid level in the PE pipe was dropped down, it means that the PE pipe was successfully inserted into the lateral ventricle. Then, after the rat was recovered from surgery, 10 μl of either SB203580 (25 μg/μl) or phosphate buffer saline (PBS, 0.01 M) was micro-infused into the lateral ventricle via a 33-gauge infusion cannula connected to a micro syringe (Gaoge, Shanghai). The drug was injected with the use of an electric microinjection pump at a flow rate of 0.5 μl/min. The cannula remained in situ for at least 5 min after infusion and was then slowly withdrawn.

### Brain dissection and tissue collection

Twenty-four hours after behavioral tests, rats (*N* = 4–6/group) from each group were anesthetized with sodium pentobarbital (150 mg/kg, i.p.) and slowly perfused with 4% paraformaldehyde (PFA). Brains were removed and post-fixed with the same fixative overnight at 4 °C followed by a graded dehydration. Brain samples were cut into serial coronal frozen sections. Sections between − 2.76 to − 4.56 mm from the bregma were selected for TUNEL, immunohistochemistry, and immunofluorescence staining.

### Immunofluorescence assay

Frozen slices (30 μm) were incubated with the primary polyclonal rabbit anti-Iba-1 (1:500), rabbit anti-GFAP (1:100), rabbit anti-NeuN (1:100), or rabbit anti-4-HNE (1:100) followed by the fluorescent-conjugated secondary antibody (goat anti-rabbit IgG, 1:200; Sigma-Aldrich). Sections were then washed three times with PBS and incubated with 4′, 6-diamidino-2-phenylindole dihydrochloride (DAPI) (Thermo Fisher Scientific, USA) at room temperature for 7 min. Images were acquired with the use of a scanning laser confocal microscope (LSM780, Carl Zeiss, Germany). At least six to eight representative images were taken from each rat for analysis by Image-Pro plus 6.0 software.

### Western blot analysis

Twenty-four hours after behavioral tests, rats were anesthetized with sodium pentobarbital (150 mg/kg, i.p.) and CA1 regions were carefully dissected for immunoblots. Briefly, CA1 was homogenized in ice-cold RIPA lysis buffer with a cocktail of protease/phosphatase inhibitors. The homogenate was centrifuged at 14,000×*g* for 10 min at 4 °C, and supernatants were collected. BCA Protein Assay Kits were used for determination of total protein concentration. Equal amounts of protein (30 μg) were loaded on SDS-PAGE gels for electrophoretic separation and then transferred onto PVDF membranes. Membranes were blocked in 5% nonfat milk for 1 h and then incubated overnight at 4 °C with the appropriate primary antibodies including polyclonal rabbit anti-βCaMKII (1:1000), polyclonal rabbit anti-phospho-ATF2 (1:500), polyclonal rabbit anti-COX-2 (1:1000), polyclonal rabbit anti-phospho-CREB (1:1000), polyclonal rabbit anti-p38 MAPK (1:1000), anti-phospho-p38 MAPK (1:500), and anti-β-actin (1:8000). The membranes were incubated with secondary horseradish peroxidase-conjugated antibodies (1:5000, Santa Cruz Biotechnology, Santa Cruz, CA) at room temperature for 1 h. Blots were detected using an enhanced chemiluminescence kit (ECL; GE Healthcare, Buckinghamshire, UK). Protein band densities were quantified using Image-J software and were normalized to β-actin.

### Reverse transcription PCR and real-time quantitative PCR

#### Reverse transcription PCR (RT-PCR)

Total RNA was extracted from samples of CA1 using a TRIpure Reagent kit (Invitrogen, USA) and reverse-transcribed into cDNA with use of the HIScriptIIQ RT SuperMix for qPCR (+gDNA wiper) kit (TaKaRa, Japan) according to the manufacturer’s instructions. cDNA for IL-1β, IFN-γ, TNF-α, and GAPDH were subsequently amplified by PCR with specific primers (Additional file 2: Table S1). PCR products were assessed by electrophoresis on a 3% agarose gel and were analyzed using the Gel Image Analysis System (Bio-rad, USA). Levels of targeted mRNA were normalized to the housekeeping gene, GAPDH.

#### Real-time quantitative PCR

Real-time PCR was performed using ChamQ SYBR qPCR Master Mix (TaKaRa, Japan) according to the manufacturer’s instructions. The primers used for qPCR are listed in Additional file 2: Table S1. GAPDH served as a loading control in each sample, and targeted gene expression levels were evaluated using the 2− (ΔΔCt) method.

### PGE2 concentration

Concentrations of PGE2 were measured using commercial assay kits (No.ml003036, Enzyme-linked Biotechnology Co. Shanghai, China) according to the manufacturer’s instructions. Total protein isolated from CA1 tissue samples was quantified using the BCA assay (Thermo Fisher, Waltham, MA). Equal amounts of diluted homogenates were added to 96-well plates. All samples were assayed in duplicate, and PGE2 levels were normalized to the total protein content.

### Statistics

Data were analyzed with SPSS version 13.0 (SPSS Inc., Chicago, IL, USA). Statistical significance of differences among groups was evaluated by one-way analysis of variance (ANOVA) followed by the Tukey’s test for multiple post hoc comparisons of means. The data were expressed as mean ± SEM. A *P* value < 0.05 was required for differences to be considered significant.

## Results

### COX-2 mediated inflammatory responses in the CA1 hippocampus in the rat model of depression

To confirm an involvement of COX-2 in this rat model of depression, we first examined the expression of COX-2 in these two stress paradigms. An overall statistically significant difference was obtained for the protein expression [*F*_(3, 20)_ = 13.82; *P* < 0.05, Fig. 1a] and mRNA expression [*F*_(3, 20)_ = 14.17; *P* < 0.05, Fig. 1b] of COX-2 among these groups. Post hoc analysis indicated that the rats subjected to CUMS-induced depression showed a significant increase in COX-2 protein levels in hippocampal CA1 regions (*P* = 0.0271). Quantitative real-time PCR revealed an increase in COX-2 mRNA levels within the CA1 region of CUMS-exposed rats (*P* = 0.0346). Levels and activity of COX-2 were also significantly increased in the LPS-induced stress paradigm. The expression of PGE2, a principal component of COX-2 products, was also significantly changed in these two stress paradigms [CUMS: *F*_(3, 20)_ = 13.57; *P* < 0.05; LPS: *F*_(3, 20)_ = 14.63; *P* < 0.05], while chronic treatment with celecoxib, which reversed the depressive phenotypes of stressed rats, resulted in a significant downregulation of PGE2 within the CA1 area of stressed rats (CUMS: *P* = 0.0359; LPS: *P* = 0.0233, Fig. 1c). These results provide substantiating evidence for an involvement of COX-2 in this rat model of depression.

As PGE2 mainly functions as a crucial pro-inflammatory factor, we further examined the potential for glial activation in the CA1 region. Our results showed that the number of Iba-1-positive cells in the CA1 region was significantly different among these groups [*F*_(3, 20)_ = 17.82; *P* < 0.001]; post hoc analysis indicates microglia activation was significantly increased in CUMS as compared to non-stressed rats (*P* = 0.0008, Fig. 1d, e). CUMS exposure also increased the number of GFAP-positive astroglia (*P* = 0.0007, Fig. 1d, f), indicating that CUMS exposure induced a significant activation of astroglial responses. Moreover, CUMS exposure significantly increased mRNA levels of IL1β [*F*_(3, 20)_ = 16.87; *P* < 0.001], TNF-α [*F*_(3, 20)_ = 16.15; *P* < 0.001], and IFN-γ [*F*_(3, 20)_ = 17.23; *P* < 0.001] within the CA1 region (Fig. 1g). Celecoxib or fluoxetine treatment significantly reversed these glial activations in stressed rats (IL1β: *P* = 0.0007; TNF-α: *P* = 0.0006; IFN-γ: *P* = 0.0009, for both). These results indicate that CUMS exposure produces a substantial neuro-inflammatory response within the CA1 area, while COX-2 blockage can significantly suppress this effect.

### The COX-2 selective inhibitor, celecoxib, ameliorates depression-like behaviors in the rat model of depression

#### Sucrose preference test

**Fig. 2:**
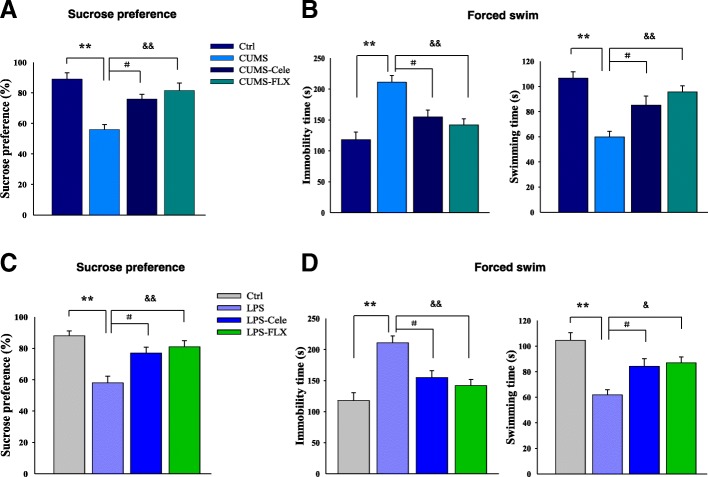
Celecoxib ameliorated depression-like behaviors in a rat model of depression. **a** Treatment of celecoxib (20 mg/kg) or fluoxetine (40 mg/kg) reversed the decreases in percent of sucrose consumption of CUMS rats. **b** Treatment of celecoxib (40 mg/kg) reversed the increases in immobility times and decreases in swimming times of CUMS rats in the forced swim test. **c** Treatment of celecoxib (40 mg/kg) reversed the decreases in percentage of sucrose consumption of rats subjected to LPS-induced depression. **d** Treatment of celecoxib (40 mg/kg) reversed the increases in immobility times and decreases in swimming times of rats subjected to LPS-induced depression. All values are presented as means ± SEM (*N* = 18). *P < 0.05, **P < 0.01, ***P < 0.001 CUMS (or LPS) vs control group; ^#^*P* < 0.05, ^##^*P* < 0.01, ^###^*P* < 0.001 CUMS (or LPS)+Cele vs depression group; ^&^*P* < 0.05, ^&&^*P* < 0.01, ^&&&^*P* < 0.001 CUMS (or LPS)+FLX vs depression group (Cele, celecoxib; FLX, fluoxetine)

Results of the sucrose preference test showed that the percentage of sucrose consumption was significantly different among these groups [*F*_(3, 68)_ = 15.26; *P* < 0.01, Fig. 2a]. Post hoc analysis indicated this after 5 weeks of CUMS exposure as compared with that of the non-stressed control group (*P* = 0.0071). Such reductions in sucrose consumption suggest that these rats are experiencing anhedonia, the loss of pleasure associated with sucrose consumption. Significant increases in sucrose consumption were observed in CUMS rats treated with the selective inhibitor of COX-2, celecoxib, (*P* = 0.0283), or the classic antidepressant, fluoxetine, (*P* = 0.0082) as compared with untreated CUMS rats. No statistically significant differences were obtained between the celecoxib-treated and fluoxetine-treated CUMS rats with regard to the percentage of sucrose consumption (*P >* 0.05).

We further examined the potent antidepressant-like effect of celecoxib within another model of depression model as achieved by treatment with LPS [13, 14]. With this model, we found that sucrose consumption in this model was also significantly changed [*F*_(3, 68)_ = 14.89; *P* < 0.01, Fig. 2c]; post hoc analysis indicated that at 1 week after LPS injection, sucrose consumption was significantly decreased (*P* = 0.0079). Again, chronic treatment of celecoxib (*P* = 0.0389) or fluoxetine (*P* = 0.0086) significantly increased sucrose consumption in these LPS-treated rats.

#### Forced swim test

In another behavioral test used to assess antidepressant-like effects, the forced swim test, rats showed significantly different immobility [*F*_(3, 68)_ = 15.16; *P* < 0.01] and swimming durations [*F*_(3, 68)_ = 14.73; *P* < 0.01] (Fig. 2b). Post hoc analysis indicated that after 5 weeks of CUMS exposure, there are increased immobility times (*P* = 0.0079) and decreased swimming times (*P* = 0.0063) as compared to the non-stressed control group. Such responses reflect behavioral despair, a core symptom of depression. In contrast, chronic treatment of celecoxib (*P* = 0.0218) or fluoxetine (*P* = 0.0076) significantly ameliorated these behavioral changes induced by CUMS exposure. Similarly, significant increases in immobility times (*P* = 0.0084) and decreases in swimming times (*P* = 0.0271) were observed in response to LPS-induced depression, while chronic treatment of celecoxib or fluoxetine exerted antidepressant-like effects in this LPS-induced stress paradigm (Fig. 2d).

Taken together, the results of these behavioral assays of depression, demonstrating that the selective inhibitor of COX-2, celecoxib, ameliorates responses associated with depression, suggest that at least part of the induction of depression phenotypes is due to COX-2 participation.

### βCaMKII is upregulated in the CA1 hippocampus in the rat model of depression

**Fig. 3:**
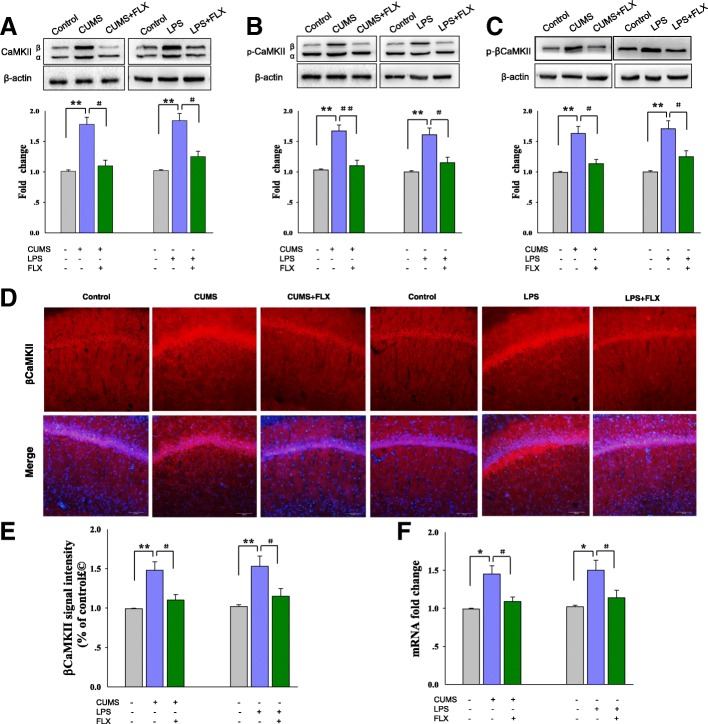
βCaMKII is upregulated in CA1 regions in a rat model of depression. **a** Western blot analysis showing protein levels of CaMKII in CA1 of rats. **b** The phosphorylation levels of CaMKII in CA1 of rats. **c** The phosphorylation levels of βCaMKII in CA1 of rats. **d** Immunofluorescence analysis of βCaMKII in CA1 of two stress paradigms. Nuclei (blue) are stained with DAPI. Scale bar is 50 μm. **e** Histograms showing the fluorescence intensities of βCaMKII in CA1 of each group. **f** QPCR analysis of βCaMKII mRNA in CA1 of two stress paradigms. *N* = 6 per group. Data are presented as the means ± SEM. *P < 0.05, **P < 0.01, ***P < 0.001 CUMS (or LPS) vs control group; ^#^*P* < 0.05, ^##^*P* < 0.01, ^###^*P* < 0.001 CUMS (or LPS)+Cele vs depression group; ^&^*P* < 0.05, ^&&^*P* < 0.01, ^&&&^*P* < 0.001 CUMS (or LPS)+FLX vs depression group (Cele, celecoxib; FLX, fluoxetine)

To investigate the main factors that trigger COX-2 enzymatic hyperactivity, we first examined potential changes in βCaMKII as associated with depression. Western blot analysis confirmed that CUMS rats showed a significant increase in βCaMKII protein [*F*_(3, 20)_ = 16.35; *P* = 0.0059] and phosphorylation levels [*F*_(3, 20)_ = 16.35; *P* = 0.0067] within the CA1 region (Fig. 3a–c). There were no significant differences between the CUMS rats and non-stressed control rats with regard to the expression of αCaMKII (*P >* 0.05). Quantitative real-time PCR also revealed an increase in βCaMKII mRNA levels in CA1 of CUMS rats [*F*_(3, 20)_ = 16.35; *P* = 0.0259], Fig. 3f). The amounts of protein [*F*_(3, 20)_ = 17.41; *P* = 0.0047] and mRNA expression levels [*F*_(3, 20)_ = 15.12; *P* = 0.0082] of βCaMKII were also significantly increased in the LPS-induced stress paradigm. Moreover, immunofluorescence staining of brain slices revealed that βCaMKII protein levels were significantly increased in the hippocampal CA1 area in both of these two stress paradigms [CUMS: *F*_(3, 20)_ = 14.98; *P* = 0.0074; LPS: *F*_(3, 20)_ = 15.07; *P* = 0.0085] (Fig. 3d, e). More importantly, chronic antidepressant treatment with fluoxetine, which reversed the depression phenotypes, produced a significant downregulation of βCaMKII protein and mRNA in the CA1 of stressed rats [CUMS: *P* = 0.0249; LPS: *P* = 0.0363]. These findings demonstrate the broad-spectrum features of βCaMKII as related to depression.

### Overexpression of βCaMKII in the CA1 hippocampus resulted in depression-like behaviors in rats

**Fig. 4:**
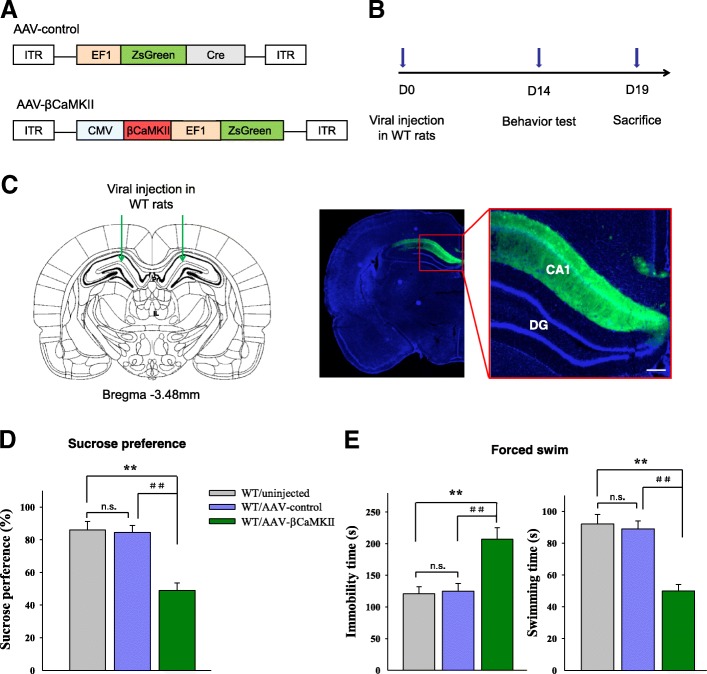
Overexpression of βCaMKII in CA1 regions produces depression-like behaviors in wild-type rats. **a** Schematics of AAV vectors engineered to overexpress a control construct or βCaMKII. ITR, inverted terminal repeats; EF1, ZsGreen promoter; CMV, βCaMKII promoter. **b** Experimental paradigm for behavioral and biochemical testing of rats infected with the virus. **c** Illustration of viral infusion of AAV-βCaMKII into the CA1 region. Scale bar is 200 μm. **d**, **e** Behavioral effects after overexpressing AAV-βCaMKII in the CA1. *N* = 18 per group. Data are presented as the means ± SEM. *P < 0.05, **P < 0.01 WT+AAV-βCaMKII vs WT; ^#^*P* < 0.05, ^##^*P* < 0.01 WT+AAV-βCaMKII vs WT+AAV-control (WT, wild type)

Based on the findings presented above, we next investigated whether hippocampal CA1 βCaMKII may serve as a key determinant in altering COX-2 function and thus the induction of depression behaviors. To examine this possibility, we constructed an AAV-βCaMKII virus which was infused bilaterally into the CA1 region of non-stressed wild-type rats for 14 days as a means to overexpress βCaMKII (Fig. 4a–c). After estimating the overexpression efficiency (*P* = 0.0006, Additional file3: Figure S2), behavioral testing for depression was then conducted. Overexpression of βCaMKII within the CA1 region of unstressed rats produced significant increases in depression-like behaviors as indicated by decreased sucrose consumption in the sucrose preference test [*F*_(3, 20)_ = 16.98; *P* = 0.0066, Fig. 4d], as well as increased immobility [*F*_(3, 20)_ = 15.75; *P* = 0.0073] and decreased swimming times [*F*_(3, 20)_ = 15.75; *P* = 0.0067] in the forced swim test as compared with rats receiving an AAV-vector control injection (Fig. 4e). These results demonstrate a pivotal role of βCaMKII in the induction of depression-like behaviors.

### Overexpression of βCaMKII activated the COX-2/PGE2 pathway within the CA1 hippocampus of rats

**Fig. 5:**
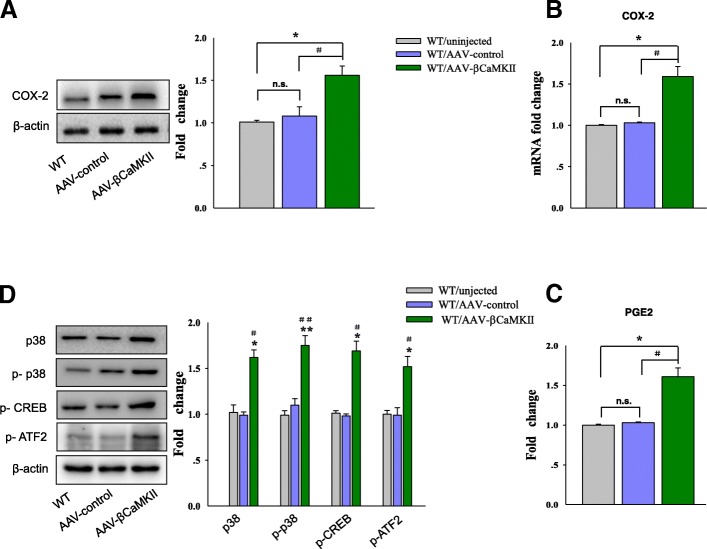
Overexpression of βCaMKII activates the COX-2/PGE2 pathway within the CA1 region of wild-type rats. **a** Western blot analysis of COX-2 protein levels in CA1 regions of rats infected by AAV-βCaMKII. **b** QPCR analysis of COX-2 mRNA in CA1 regions of rats infected by AAV-βCaMKII. **c** Content assays showing expressions of PGE2 in CA1 regions of rats infected by AAV-βCaMKII. **d** Western blot analysis of p38, p-p38, p-CREB, and p-ATF2 expression levels in CA1 regions of rats infected by AAV-βCaMKII. *N* = 6 per group. Data are presented as the means ± SEM. *P < 0.05, **P < 0.01 WT+AAV-βCaMKII vs WT; ^#^*P* < 0.05, ^##^*P* < 0.01 WT+AAV-βCaMKII vs WT+AAV-control (WT, wild type)

To determine some of the potential downstream targets of βCaMKII, we first assessed expressions of COX-2 and PGE2 in CA1 regions after AAV-βCaMKII injection. βCaMKII overexpression produced an overall statistically significant increase in both the protein [*F*_(2, 15)_ = 12.94; *P* = 0.0313, Fig. 5a] and mRNA expressions [*F*_(2, 15)_ = 13.25; *P* = 0.0283, Fig. 5b] of COX-2, as well as an increase in the production of PGE2 [*F*_(2, 15)_ = 14.23; *P* = 0.0277, Fig. 5c].

Such findings indicate that βCaMKII may be a likely candidate responsible for PGE2 production, which is induced by stress exposure in this rat model of depression. To substantiate this result, we next examined the modulatory effects of βCaMKII on potential proteins related to the activation of COX-2. As shown in Fig. 5d, both the protein [*F*_(2, 15)_ = 12.86; *P* = 0.0214] and phosphorylation levels [*F*_(2, 15)_ = 15.18; *P* = 0.0077] of p38 MAPK were significantly increased within the CA1 area of the AAV-βCaMKII injection group. Moreover, as compared to that of rats receiving an AAV vector-control injection, statistically significant increases were obtained for phosphorylation levels of potential transcription factors related to COX-2, such as CREB [*F*_(2, 15)_ = 14.11; *P* = 0.0135] and ATF-2 [*F*_(2, 15)_ = 14.82; *P* = 0.0169] within CA1 regions.

### Knocking down of βCaMKII in CA1 hippocampus rescued depression-like behaviors in rats

**Fig. 6:**
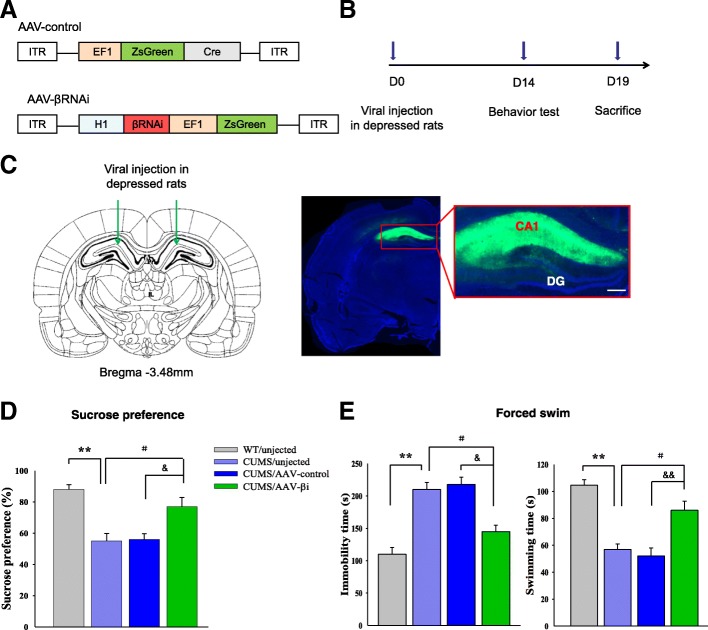
Knocking down of βCaMKII in CA1 rescued depression-like behaviors of depressed rats. **a** Schematics of AAV vectors engineered to overexpress a control construct or RNAi form of βCaMKII. H1, human H1 promoter. **b** Experimental paradigm for behavioral testing of rats infected by virus. **c** Illustration of viral infusion of βCaMKII RNAi construct into the CA1. Scale bar is 200 μm. **d**, **e** Behavioral effects after expressing AAV-βCaMKII RNAi construct within the CA1 of depressed rats. *N* = 18 per group. Data are presented as the means ± SEM. *P < 0.05, **P < 0.01 CUMS+AAV-βi vs CUMS; ^#^*P* < 0.05, ^##^*P* < 0.01 CUMS+AAV-βi vs CUMS+AAV-control (AAV-βi, AAV-βCaMKII RNAi)

The goal of our next experiment was to determine whether a downregulation of βCaMKII or blockade of βCaMKII function within the CA1 region would rescue the display of depression phenotypes. To accomplish this goal, we constructed an AAV virus which expressed the RNA interference (RNAi) form of βCaMKII (AAV-βRNAi) to knock down βCaMKII protein (Fig. 6a) and infused it bilaterally into the CA1 region of depressed rats (Fig. 6c). After estimating the knockdown efficiency (*P* = 0.0008, Additional file 4: Figure S3), behavioral testing of depression was then performed at 14 days post-infusion of the AAV-βRNAi (Fig. 6b). A statistically significant amelioration was observed in depression-like behavioral responses as indicated by increases in sucrose consumption [*F*_(3, 20)_ = 12.49; *P* = 0.0273, Fig. 6d], decreases in immobility times [*F*_(3, 20)_ = 13.20; *P* = 0.0291], and increases in swimming times [*F*_(3, 20)_ = 13.96; *P* = 0.0319, Fig. 6e] in rats infected with AAV-βRNAi as compared with depressed rats receiving the AAV vector-control injection. These behavioral results provide further evidence that an upregulation of βCaMKII contributes to depression-like behaviors induced by stress exposure, while downregulation of βCaMKII within the CA1 region ameliorated these symptoms.

### Knocking down of βCaMKII in CA1 hippocampus suppressed hyperactivity of the COX-2/PGE2 pathway in depressed rats

**Fig. 7:**
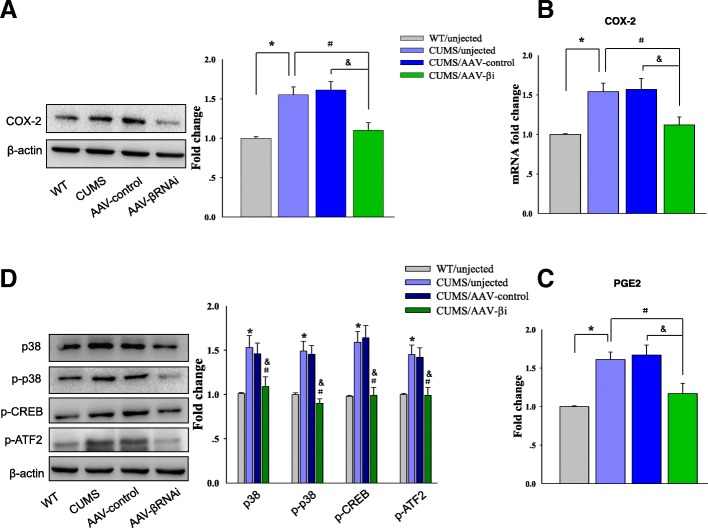
Knocking down of βCaMKII in CA1 suppresses hyperactivity of the COX-2/PGE2 pathway in depressed rats. **a** Western blot analysis of COX-2 protein levels in CA1 infected by AAV-βCaMKII RNAi. **b** QPCR analysis of COX-2 mRNA in CA1 regions. **c** Content assays showing expressions of PGE2 in CA1 regions. **d** Western blot analysis of p38, p-p38, p-CREB, and p-ATF2 expression levels in CA1 regions. *N* = 6 per group. Data are presented as the means ± SEM. *P < 0.05, **P < 0.01 CUMS+AAV-βi vs CUMS; ^#^*P* < 0.05, ^##^*P* < 0.01 CUMS+AAV-βi vs CUMS+AAV-control (AAV-βi, AAV-βCaMKII RNAi)

In addition to its effects on depression-like behaviors, knocking down of βCaMKII also substantially modulated molecular markers of the COX-2/PGE2 pathway. Specifically, protein [*F*_(3, 20)_ = 13.83; *P* = 0.0301, Fig. 7a] and mRNA [*F*_(3, 20)_ = 14.02; *P* = 0.0280, Fig. 7b] expressions of COX-2 within the CA1 area were significantly decreased at 14 days after AAV-βRANi injection in depressed rats. As a result, the expression of PGE2 was also significantly decreased as compared to that of depressed rats injected with the AAV-vector control [*F*_(3, 20)_ = 12.89; *P* = 0.0327, Fig. 7c].

Moreover, protein expressions of p38 MAPK [*F*_(3, 20)_ = 15.07; *P* = 0.0080] and phosphorylation levels related to p38 MAPK [*F*_(3, 20)_ = 15.23; *P* = 0.0076], CREB [*F*_(3, 20)_ = 16.71; *P* = 0.0058], and ATF-2 [*F*_(3, 20)_ = 14.97; *P* = 0.0086] within CA1 regions were also significantly decreased in the AAV-βRNAi injection group (Fig. 7d). Any or all of these factors may be responsible for the transcriptional downregulation of COX-2 and thus the reduced production of PGE2.

These results provide evidence indicating that suppression of phosphorylated p38 MAPK may contribute to the antidepressant-like behaviors resulting from downregulation of βCaMKII.

### The p38 MAPK antagonist SB203580 reduced activity of the COX-2/PGE2 pathway and depression-like behaviors

**Fig. 8:**
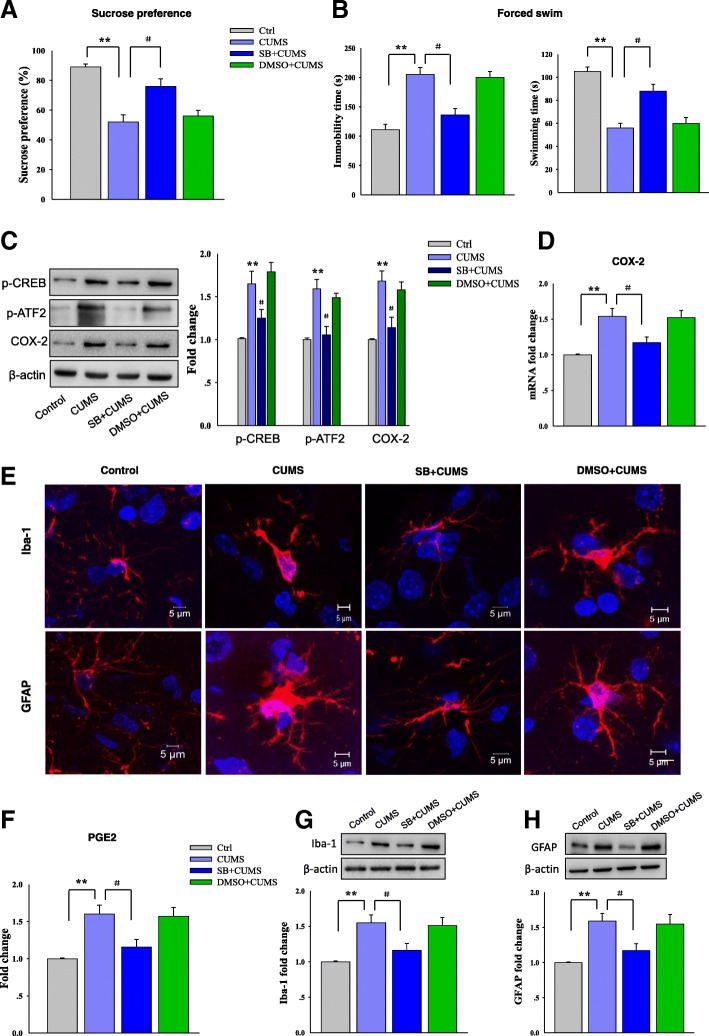
Blocking p38 MAPK reduces activity of the COX-2/PGE2 pathway and depression-like behaviors in rats. **a**, **b** Pretreatment of SB203580 (10 μg/kg) ameliorated the depression-like behaviors in CUMS rats. **c** Pretreatment of SB203580 reversed the decreased phosphorylation levels of CREB, ATF2, and COX-2 expression in CA1 caused by CUMS exposure. **d** SB203580 attenuated the increased COX-2 mRNA levels in CA1 resulting from CUMS exposure. **e** SB203580 reduced the morphological changes of microglia and astrocytes within the CA1 resulting from CUMS exposure. **f** SB203580 attenuated the increased PGE2 content levels in CA1 resulting from CUMS exposure. **g** SB203580 reduced the increased Iba1 protein levels within the CA1 resulting from CUMS exposure. **h** SB203580 reduced the increased GFAP protein levels within the CA1 resulting from CUMS exposure. *N* = 6 per group. Data are presented as the means ± SEM. *P < 0.05, **P < 0.01, ***P < 0.001 CUMS vs control group; ^#^*P* < 0.05, ^##^*P* < 0.01, ^###^*P* < 0.001 CUMS+SB203580 vs CUMS group (SB, SB203580)

Next, to determine whether phosphorylation of the p38 MAPK pathway may contribute to the activation of COX-2/PGE2 and depression in rats, we injected SB203580 to block p38 MAPK activity prior to CUMS exposure. Our behavioral results showed that SB203580 significantly reversed the decreased sucrose consumption [*F*_(3, 20)_ = 12.97; *P* = 0.0228], the increased immobility [*F*_(3, 20)_ = 14.31; *P* = 0.0187], and decreased swimming times [*F*_(3, 20)_ = 13.57; *P* = 0.0216] produced by CUMS exposure (Fig. 8a, b). In addition, SB203580 injection significantly suppressed CREB [*F*_(3, 20)_ = 14.17; *P* = 0.0251] and ATF-2 [*F*_(3, 20)_ = 12.77; *P* = 0.0360] phosphorylation levels as compared to the CUMS alone exposure group (Fig. 8c). The increased protein [*F*_(3, 20)_ = 13.08; *P* = 0.0316, Fig. 8c] and mRNA [*F*_(3, 20)_ = 14.22; *P* = 0.0207] expressions of COX-2 (Fig. 8d), as well as the increased content of PGE2 [*F*_(3, 20)_ = 14.53; *P* = 0.0197, Fig. 8f] within CA1 regions resulting from CUMS exposure, were also reversed by SB203580 injection. We also examined CA1 inflammatory response in response to this treatment. Immunofluorescence assays revealed that SB203580 significantly ameliorates the rounded amoeboid-like appearance of microglia and cellular hypertrophy of astrocytes (Fig. 8e), as well as reduced the expression levels of Iba-1 [*F*_(3, 20)_ = 13.82; *P* = 0.0327, Fig. 8g] and GFAP [*F*_(3, 20)_ = 13.09; *P* = 0.0392, Fig. 8h] in the CA1 region as compared to non-SB203580-treated CUMS rats.

Taken together with results obtained from the AAV virus injection experiments, these results indicate that p38 MAPK may be a critical downstream molecular target of βCaMKII in modulating the COX-2/PGE2 pathway and behavioral responses related to depression.

## Discussion

**Fig. 9:**
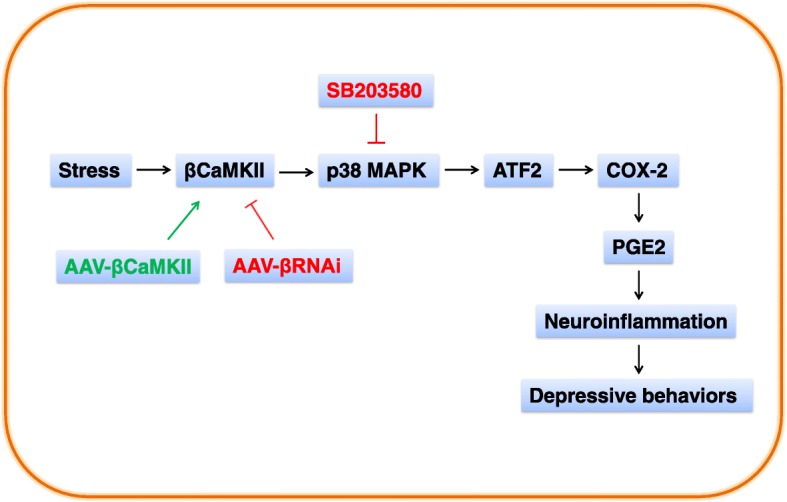
Proposed role of βCaMKII in regulation of COX-2/PGE2 neuroinflammatory signaling pathway and depression-like behaviors. Chronic stress upregulates βCaMKII expression and activates p38 MAPK, with subsequent activation of ATF2 and transcription of COX-2. This causes increased PGE2 synthesis and neuroinflammation, which eventually induced depressive behaviors. AAV-βCaMKII overexpressed βCaMKII, to prompt this signaling pathway. AAV-βRNAi induced knockdown of βCaMKII, to suppress this pathway. SB203580 treatment prevented p38 MAPK activation and thus attenuated the inflammation and depressive behaviors

Depression is a highly heterogeneous disease characterized by diversity of behavioral symptoms, which constantly develop into a diversity of potential therapeutic treatments. For example, deep brain stimulation was recently demonstrated a valuable therapeutic intervention, through which exerts antidepressant effects at least partially via modulating the monoamine neurotransmitters in target regions and the activity of interconnected brain networks of treatment-resistant depression [16]. Meanwhile, neuroinflammation is also considered a critical factor in the etiology of depression [17, 18]. However, the pathophysiological mechanisms are not fully understood. In the present study, our results showed that stress-induced upregulation of βCaMΚΙΙ in the CA1 region results in phosphorylation of p38 MAPK. As a result, this enhances the transcriptional function of the CREB/ATF-2 family and hence prompts COX-2 transcription and expression. The upregulation of COX-2 may augment the production of its downstream molecule, PGE2, in CA1 neurons. This orchestrated cascade of events may then induce a hyperactivity in inflammatory responses and thus lead to behavioral depression in rats (Fig. 9).

Recent findings from clinical studies have indicated that elevated microglia activation in specific brain regions is associated with depression in patients who are inclined to commit suicide [19, 20]. Interestingly, accumulating evidence has revealed that some antidepressants, such as the classic selective serotonin reuptake inhibitor (SSRI), fluoxetine, exert an anti-inflammatory effect via downregulating microglial activation [21]. A previous study suggested that fluoxetine modulated the immune system by inhibiting M1 activation and by improving M2 activation of microglia [22]. Further studies showed that fluoxetine diminished the nuclear translocation of nuclear factor kappaB (NF-κB) p65 subunit and inhibits the LPS-induced glia activation and expression of TNF-α and IL-1β [23]. Such a normalization of inflammatory markers is often accompanied with remission of depressive symptoms in some patients [24, 25]. All of these observations suggest that targeting the neuroinflammatory system to develop new therapeutic strategies for the treatment of depression might provide a novel and valuable route for future research in this area. Microglial reactivity was found in the hippocampus of rodents following exposure to chronic unpredictable stress and LPS-induced depressive-like behavior [26, 27], while suppressing LPS-induced microglia activation and pro-inflammatory cytokine production in the hippocampus could significantly attenuate the neuroinflammation and depressive-like behaviors [28–31]. In response to stressful conditions, microglial cells have the capacity to change their morphology from a surveillance phenotype to one of an enlarged soma and retracted processes [32, 33], effects which, together with astrocytes, are usually associated with the production of pro-inflammatory cytokines [34]. Consistent with these studies, our data are in general agreement with the findings that fluoxetine improved behavioral responses in two animal models of depression, by attenuating the activation of microglia and astrocytes within the CA1 hippocampus, one of the key brain areas associated with MDD and other psychiatric disorders. These results suggest that activation of glial cell responses may contribute to the genesis and development of depression conferred by stress.

Of particular significance with regard to the involvement of these inflammatory mechanisms, our current results indicate that pretreatment with celecoxib, as a means to block COX-2 activity, inhibited the stress-induced glial activation and depression-like behaviors in rats. Such an effect likely involves an inhibition of COX-2 enzymatic activity and the subsequent pro-inflammatory factor, PGE2. These observations support the concept that a reciprocal relationship exists between the effects of antidepressants and neuroinflammatory responses which may then, in part, contribute to the behavioral alterations underlying depression. In this way, we provide new evidence that links Cox-2 overexpression with PGE2 production and the subsequent activation of microglia and astrocytes in an animal model of depression. Such results suggest that activation of the COX-2/PGE2-mediated inflammatory pathway may represent a significant component in the pathophysiology of depression. Unfortunately, as COX-2 antagonists are usually accompanied with significant cardiovascular and gastrointestinal side effects, it will be necessary to identify new targets within the COX-2-mediated neuroinflammatory system to develop effective and safe therapeutic strategies for the treatment of depression.

A previous study found that βCaMKII expression was significantly upregulated in the lateral habenula, another key brain region in the pathophysiology of depression, in animal models of depression and was downregulated by antidepressants [9]. In addition, βCaMΚΙΙ is also highly concentrated in the hippocampus [35]. Moreover, it is more sensitive to calcium/calmodulin than other isoforms and has an actin-binding site that is absent in aCaMKII [36]. This high expression and specialized features of βCaMΚΙΙ likely confer specific functions of this molecule within the hippocampus that distinguishes it from those of other isoforms. In this study, we found that βCaMΚΙΙ expression was significantly upregulated in the CA1 region in a rat model of depression while downregulated in response to treatment with the antidepressant, fluoxetine. Furthermore, overexpression of βCaMΚΙΙ in the CA1 region of wild-type rats via AAV-virus injection effectively induced depression-like behaviors and strongly increased the phosphorylation levels of p38 MAPK and the transcription factors CREB and ATF-2. Results from previous studies have indicated that nicotine-triggered Ca^2+^ leads to a transient activation of CaMKII and sequential phosphorylation of p38 MAPK in PC12 cells [37]. In endothelial cells, inhibition of the Ca^2+^ downstream target βCaMΚΙΙ suppressed the phosphorylation levels of p38 MAPK [38]. In addition, the transcription factor, ATF-2, is also a target of the p38 MAPK and SAPK/JNK kinase signaling pathways and can thus serve as a crossroad between nuclear and cytosolic functions [39]. ATF-2 usually binds to the cAMP response element (CRE)-containing DNA response elements and is a vital member of the CREB/ATF family [40]. Therefore, the results from our present study suggest that exposure to chronic stress might upregulate βCaMΚΙΙ in CA1regions, which then phosphorylates p38 MAPK and subsequently activates the ATF-2/CREB family to enhance their transcription function.

More importantly, we demonstrate here that an upregulation of βCaMΚΙΙ in CA1 significantly enhanced the activity of the COX-2/PGE2 pathway and was sufficient to produce a distinct array of core symptoms of depression. Conversely, knocking down βCaMΚΙΙ in CA1 with the use of an AAV-βRNAi injection significantly reversed these changes in biochemical parameters, along with a rescue of depression symptoms. Recent studies have indicated that the p38 MAPK/ATF-2 signaling pathway induces COX-2-derived PGE2 production during inflammatory responses in brain injury and in inflammatory diseases [41]. Accordingly, ATF-2 may potentially function as a critical molecular target for drug therapies in neurodegenerative diseases [42]. Taken together, our data demonstrate that a Ca^2+^-dependent βCaMΚΙΙ response appears to provide a bridge between stress exposure and neuroinflammatory activation. In this way, the linking of p38 MAPKs and CREB/ATF2-induced COX-2 upregulation by βCaMKII might be a key factor in the pathogenesis of this psychiatric disorder.

Our results showed that exposure to chronic stress or an overexpression of βCaMΚΙΙ in CA1 enhances COX2-induced neuroinflammation and depression-like behaviors in rats, likely via enhancing p38-mediated transcription activity and subsequent induction of pro-inflammatory molecules. In contrast, knocking down of βCaMΚΙΙ as achieved with the use of an AAV-βRNAi injection may downregulate the activity of the p38 MAPK pathway, thus ameliorating these depressive phenotypes induced by chronic stress. These data suggest that the p38 MAPK pathway appears to be a bridge between βCaMΚΙΙ activation and neuroinflammation. It has been reported that p38 MAPK transduces signals from cell membranes to the nucleus and, in this way, is responsible for regulating a variety of cellular activities in response to certain environmental stimuli [43]. Once activated, p38 can shuttle between the cytoplasm and nucleus to regulate gene expression for adaptation to environmental changes [44]. To confirm the potential for such crosstalk roles of p38 in the pathogenesis of depression by chronic stress, we blocked the p38 MAPK activity with the use of its selective inhibitor, SB203580, during CUMS exposure. Our results showed that blocking p38 MAPK significantly downregulated the increase in CREB/ATF-2 phosphorylation levels resulting from chronic stress, which may then ameliorate the depressive phenotypes induced by ATF2-mediated COX-2/PGE2 hyperactivity. Such findings suggest that p38 may act as an upstream activator to increase the transcription activity of AFT-2. Related results from a previous study have supported these findings as p38 MAPK has been shown to phosphorylate ATF-2 at Thr69 and Thr71 as demonstrated in vitro and in cells transfected with ATF-2 [45, 46]. Moreover, p38 MAPK has been shown to represent an important signal transduction pathway in the regulation of inflammation [47]. Collating these findings, it seems likely that βCaMΚΙΙ-activated p38 MAPK cascades may participate in the generation of neuroinflammation and depression-like behaviors in animal models. In this way, βCaMΚΙΙ/p38 MAPK activation may serve as an important trigger involved in the neurobiological and behavioral changes resulting in depression. The modulatory effects of fluoxetine upon these pathways may also provide some potentially worthwhile therapeutic targets for depression.

The major question to be addressed is how depressive stimuli lead to βCaMKII expression changes in the hippocampus. It is considered that physical or aversive emotional stimuli induced hyperactivity of the hypothalamic–pituitary–adrenal (HPA) axis [48, 49]. Adrenal glucocorticoids are major modulators of multiple functions including immunity, stress responses, and cognition [50, 51]. Therefore, a new hypothesis suggested that βCaMKII levels in the hippocampus may be due to the interaction of released glucocorticoids with hippocampal receptors and thus lead to subsequent neuroinflammation [52]. However, the detailed molecular and clinical mechanisms of how corticosteroid signaling regulates βCaMKII expression need further investigation.

## Conclusion

In conclusion, the present study revealed a novel neuroprotective mechanism whereby antidepressants exert effects via preventing βCaMΚΙΙ-mediated neuronal inflammation in a rat model of depression. These results showed that a stress-induced upregulation of βCaMΚΙΙ within the hippocampal CA1 area produced an increase in the activity of p38 MAPK. As a result, there is an increase in the transcription efficiency of ATF-2, thus triggering COX2-mediated neuroinflammation which is associated with depression. Better yet, we found that manipulation of βCaMKII levels in the hippocampus CA1 could effectively modulate depressive symptoms, which suggests that βCaMKII, functioning as the upstream of this neuroinflammatory signaling pathway, might be an important potential target for novel antidepressant to control depressive symptoms. Hence, this study provides a strong foundation for the direction of new avenues of investigation in the development of effective therapeutic strategies which can identify and manage inflammation-related psychiatric disorders.

## Additional files

Additional file 1: **Figure S1.** Depression animal model experimental design: schematic figure of the treatment protocol of rats. (A) CUMS depression model paradigm. (B) LPS-induced depression model paradigm. CUMS, chronic unpredictable mild stress; i.p., intraperitoneal; Cele, celecoxib; FLX, fluoxetine; SPT, sucrose preference test; FST, forced swim test; WB, Western blot; Q-PCR, quantitative real-time PCR; RT-PCR, reverse transcription PCR; ELISA, enzyme-linked immunosorbent assay; IF, immunofluorescence. (TIF 46 kb)

Additional file 2: **Table S1.** PCR primers used in this study. (DOCX 17 kb)

Additional file 3: **Figure S2.** Estimation of βCaMKII overexpression efficiency after viral constructs unilateral injection. (A) Pictures represented coronal brain slices of rats that have received unilateral injection of AAV-βCaMKII viruses. Left: non-injected side. Right: injected side. (B) Level of overexpression represented by ratio of βCamKII fluorescence signal intensity of the injected and non-injected side. (C) Representative Western blot and quantification of βCaMKII overexpression efficiency. *N* = 6 per group. Data were presented as the means ± SEM. *P < 0.05, **P < 0.01 WT+AAV-βCaMKII vs non-injected side; ^#^*P* < 0.05. (WT, wild type). (TIF 1360 kb)

Additional file 4: **Figure S3.** Estimation of βCaMKII knockdown efficiency after viral constructs unilateral injection. (A) Pictures represented coronal brain slices of rats that have received unilateral injection of AAV-βCaMKII RNAi viruses. Left: non-injected side. Right: injected side. (B) Level of knocking down represented by ratio of βCamKII fluorescence signal intensity of the injected and non-injected side. (C) Representative Western blot and quantification of βCaMKII knockdown efficiency. *N* = 6 per group. Data were presented as the means ± SEM. *P < 0.05, **P < 0.01 CUMS+AAV-βi vs CUMS; ^#^*P* < 0.05, ^##^*P* < 0.01 CUMS+AAV-βi vs CUMS+AAV-control. (AAV-βi, AAV-βCaMKII RNAi). (TIF 1196 kb)

## Abbreviations

(COX)-2: Cyclo-oxygenase 2
4-HNE: 4-Hidroxynonenal
AAV: Adenovirus-associated virus
AD: Alzheimer’s disease
ANOVA: Analysis of variance
ATF2: Activating transcription factor 2
CaMKII: Calcium/calmodulin-dependent protein kinase type II
CNS: Central nervous system
CUMS: Chronic unpredictable mild stress
DMSO: Dimethyl sulfoxide
ELISA: Enzyme-linked immunosorbent assay
FST: Forced swim test
GFAP: Glial fibrillary acidic protein
i.p.: Intraperitoneal
IL-1: Interleukin 1
LPS: Lipopolysaccharide
MDD: Major depressive disorder
P38 MAPK: P38 mitogen-activated protein kinase
PBS: Phosphate buffer saline
PD: Parkinson’s disease
PGE2: Prostaglandin E2
RT-PCR: Reverse transcription PCR
SPT: Sucrose preference test
TNF-α: Tumor necrosis factor-α
