# Supplementary figures and images for: Hippocampal CA1 βCaMKII mediates neuroinflammatory responses via COX-2/PGE2 signaling pathways in depression
Source: J Neuroinflammation. 2018 Dec 8;15:338. doi: 10.1186/s12974-018-1377-0 (PMC6286788; doi:10.1186/s12974-018-1377-0)

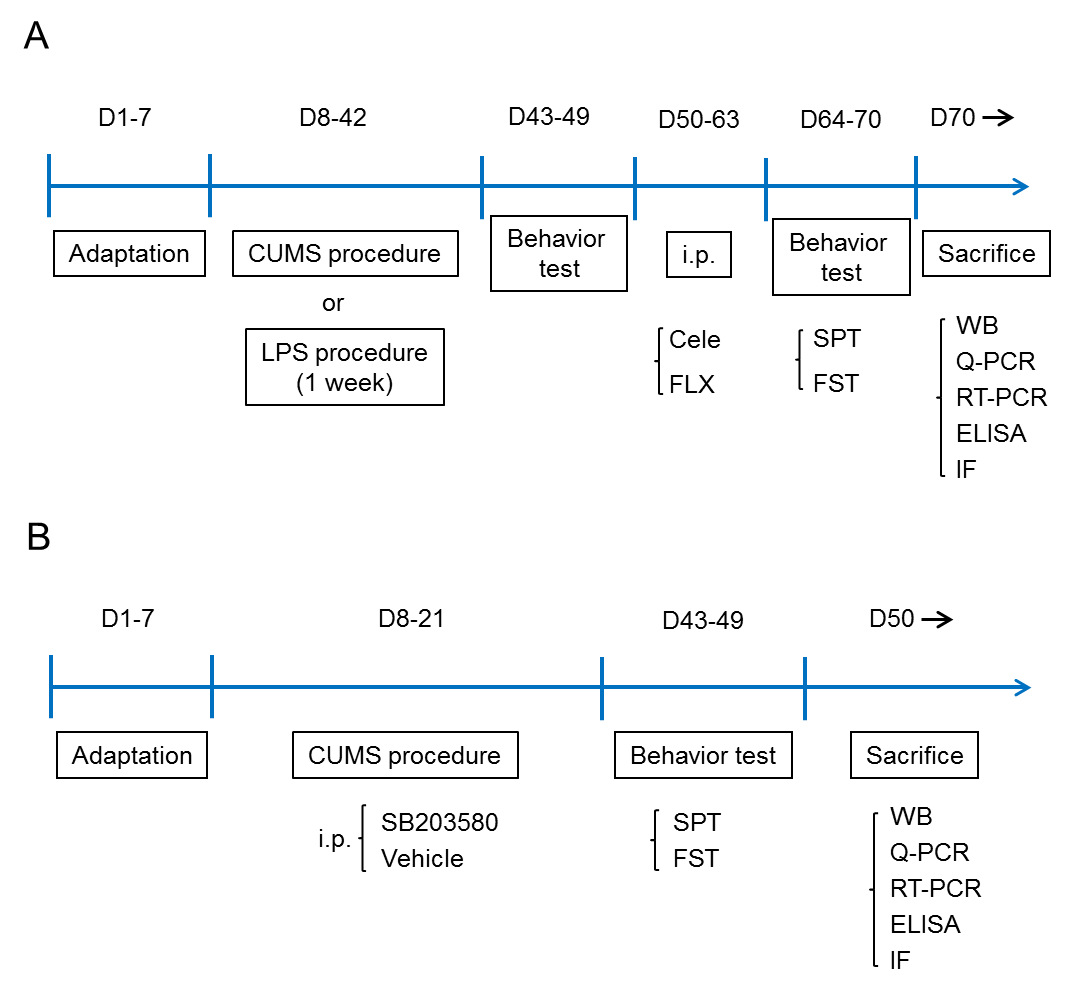

Supplement: Supplementary file 1 — Figure S1. Depression animal model experimental design: schematic figure of the treatment protocol of rats. (A) CUMS depression model paradigm. (B) LPS-induced depression model paradigm. CUMS, chronic unpredictable mild stress; i.p., intraperitoneal; Cele, celecoxib; FLX, fluoxetine; SPT, sucrose preference test; FST, forced swim test; WB, Western blot; Q-PCR, quantitative real-time PCR; RT-PCR, reverse transcription PCR; ELISA, enzyme-linked immunosorbent assay; IF, immunofluorescence. (TIF 46 kb) [file 12974_2018_1377_MOESM1_ESM.tif]

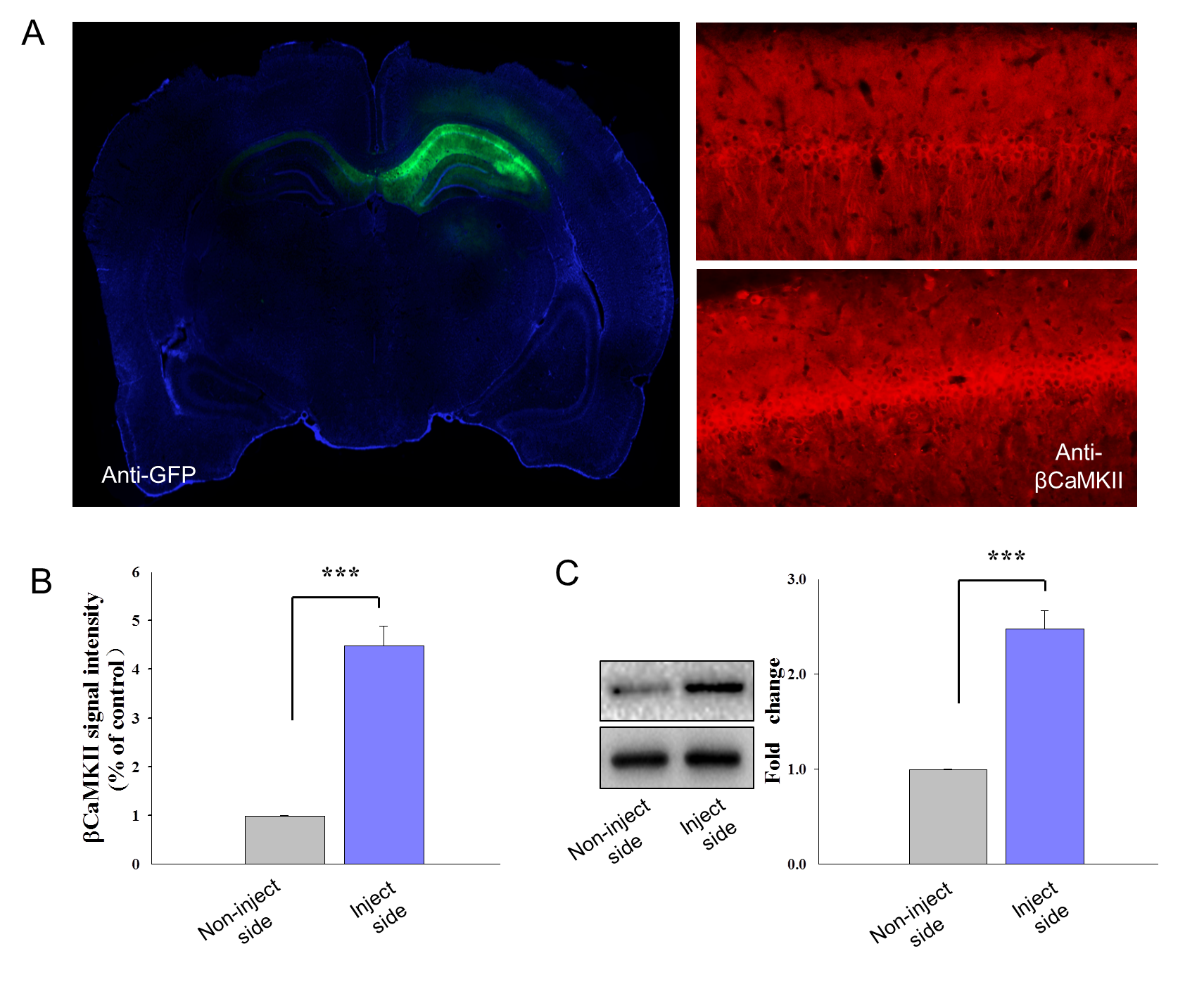

Supplement: Supplementary file 3 — Figure S2. Estimation of βCaMKII overexpression efficiency after viral constructs unilateral injection. (A) Pictures represented coronal brain slices of rats that have received unilateral injection of AAV-βCaMKII viruses. Left: non-injected side. Right: injected side. (B) Level of overexpression represented by ratio of βCamKII fluorescence signal intensity of the injected and non-injected side. (C) Representative Western blot and quantification of βCaMKII overexpression efficiency. N = 6 per group. Data were presented as the means ± SEM. *P < 0.05, **P < 0.01 WT+AAV-βCaMKII vs non-injected side; #P < 0.05. (WT, wild type). (TIF 1360 kb) [file 12974_2018_1377_MOESM3_ESM.tif]

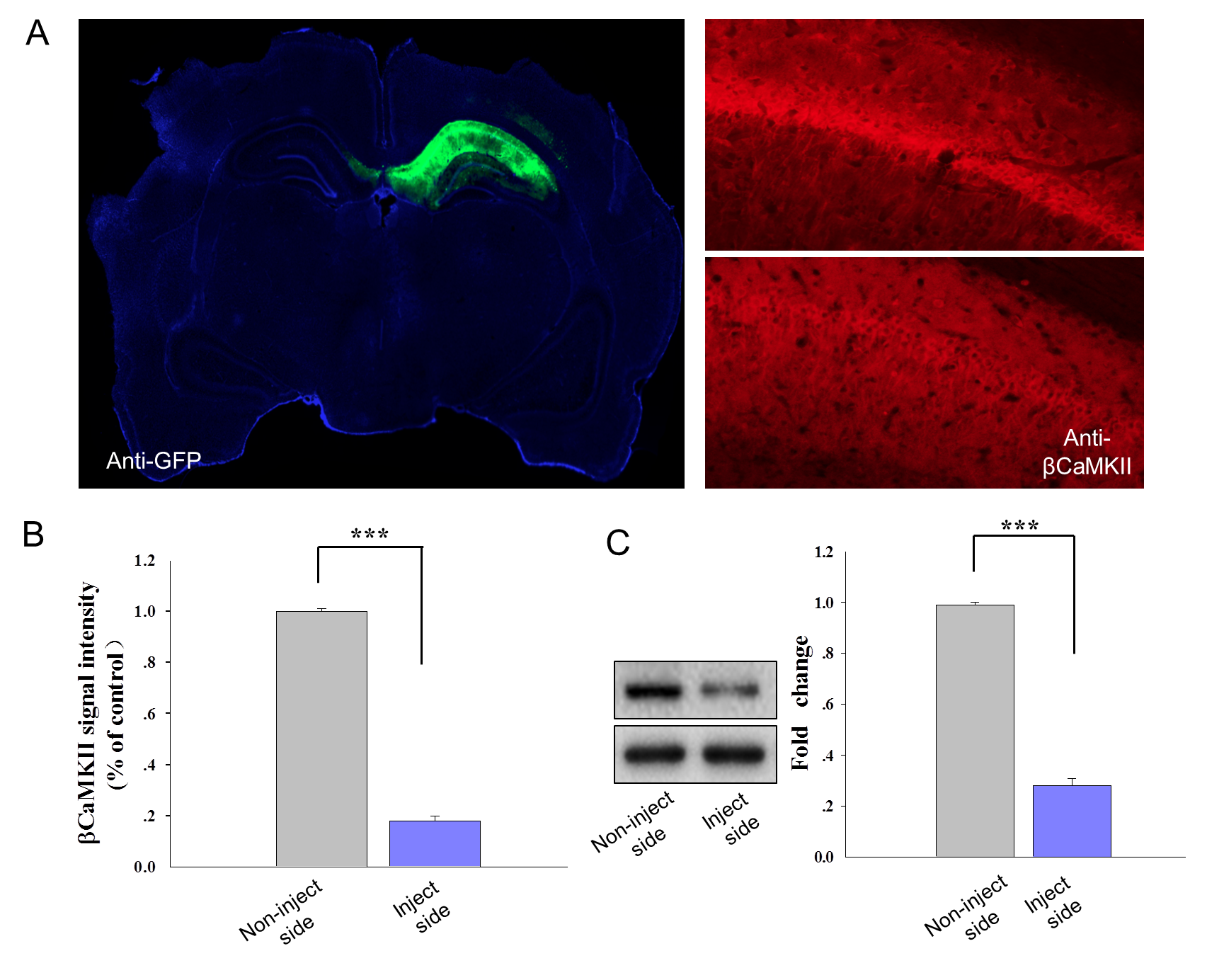

Supplement: Supplementary file 4 — Figure S3. Estimation of βCaMKII knockdown efficiency after viral constructs unilateral injection. (A) Pictures represented coronal brain slices of rats that have received unilateral injection of AAV-βCaMKII RNAi viruses. Left: non-injected side. Right: injected side. (B) Level of knocking down represented by ratio of βCamKII fluorescence signal intensity of the injected and non-injected side. (C) Representative Western blot and quantification of βCaMKII knockdown efficiency. N = 6 per group. Data were presented as the means ± SEM. *P < 0.05, **P < 0.01 CUMS+AAV-βi vs CUMS; #P < 0.05, ##P < 0.01 CUMS+AAV-βi vs CUMS+AAV-control. (AAV-βi, AAV-βCaMKII RNAi). (TIF 1196 kb) [file 12974_2018_1377_MOESM4_ESM.tif]
