# Supplementary material for: Hippocampal CA1 βCaMKII mediates neuroinflammatory responses via COX-2/PGE2 signaling pathways in depression
Source: J Neuroinflammation. 2018 Dec 8;15:338. doi: 10.1186/s12974-018-1377-0 (PMC6286788; doi:10.1186/s12974-018-1377-0)
Supplement: Supplementary file 2 — Table S1. PCR primers used in this study. (DOCX 17 kb) [file 12974_2018_1377_MOESM2_ESM.docx]

**Supplemental Table 1.** PCR primers used in this study

| *Gene* | *Forword (*5’→3’*)* | *Reverse (*5’→3’*)* |
| --- | --- | --- |
| IL-1β | AAG ATG AAG GGC TGC TTC CAA ACC | ATA CTG CCT GCC TGA AGC TCT TGT |
| IFN-γ | ATT CAT GAG CAT CGC CAA GTT C | TGA CAG CTG GTG AAT CAC TCT GAT |
| TNF-α | TGA TCG GTC CCA ACA AGG A | TGC TTG GTG GTT TGC TAC GA |
| COX-2 | TGT GAA AGG GTG TCC CTT CG | ACA ACA CAG GAA TCT TCA CAA ATG G |
| GAPDH | AGT GCC AGC CTC GTC TCA TA | GGT AAC CAG GCG TCC GAT AC |
